# α-synuclein induced synapse damage is enhanced by amyloid-β_1-42_

**DOI:** 10.1186/1750-1326-5-55

**Published:** 2010-12-07

**Authors:** Clive Bate, Steve Gentleman, Alun Williams

**Affiliations:** 1 Department of Pathology and Infectious Diseases, Royal Veterinary College, Hawkshead Lane, North Mymms, Herts., AL9 7TA, UK; 2 Neuropathology Unit, Department of Medicine, Imperial College London, Charing Cross Campus, St Dunstan's Road, London, W6 8RP, UK; 3 Department of Veterinary Medicine, University of Cambridge, Madingley Road, Cambridge, CB3 OES, UK

## Abstract

**Background:**

The pathogenesis of Parkinson's disease (PD) and dementia with Lewy bodies (DLB) is associated with the accumulation of aggregated forms of the α-synuclein (αSN) protein. An early event in the neuropathology of PD and DLB is the loss of synapses and a corresponding reduction in the level of synaptic proteins. However, the molecular mechanisms involved in synapse damage in these diseases are poorly understood. In this study the process of synapse damage was investigated by measuring the amount of synaptophysin, a pre-synaptic membrane protein essential for neurotransmission, in cultured neurons incubated with αSN, or with amyloid-β (Aβ) peptides that are thought to trigger synapse degeneration in Alzheimer's disease.

**Results:**

We report that the addition of recombinant human αSN reduced the amount of synaptophysin in cultured cortical and hippocampal neurons indicative of synapse damage. αSN also reduced synaptic vesicle recycling, as measured by the uptake of the fluorescent dye FM1-43. These effects of αSN on synapses were modified by interactions with other proteins. Thus, the addition of βSN reduced the effects of αSN on synapses. In contrast, the addition of amyloid-β (Aβ)_1-42 _exacerbated the effects of αSN on synaptic vesicle recycling and synapse damage. Similarly, the addition of αSN increased synapse damage induced by Aβ_1-42_. However, this effect of αSN was selective as it did not affect synapse damage induced by the prion-derived peptide PrP82-146.

**Conclusions:**

These results are consistent with the hypothesis that oligomers of αSN trigger synapse damage in the brains of Parkinson's disease patients. Moreover, they suggest that the effect of αSN on synapses may be influenced by interactions with other peptides produced within the brain.

## Background

Parkinson's disease (PD) is a neurodegenerative motor disorder affecting up to 2% of the population over the age of 65. Although it is characterised by the presence of bradykinesia, resting tremor and rigidity, up to 88% of patients also show significant psychiatric and autonomic symptoms [1]. The most common of these non-motor symptoms are Parkinson's disease dementia (PDD), with a cumulative prevalence ranging between 50 and 75% of cases [2] and dementia with Lewy Bodies (DLB), a similar condition to PDD except that dementia rather than motor symptoms are primary. DLB is the second most common cause of dementia after Alzheimer's disease (AD) and is characterised by progressive cognitive decline and parkinsonism [3]. Currently, there is no long-term cure for PD, PDD or DLB.

The major histopathological hallmark of PD, PDD and DLB is the alpha-synuclein (αSN) positive intraneuronal inclusion known as a Lewy body (LB). Although the presence of LBs in the substantia nigra is diagnostic for PD, αSN pathology is also seen in multiple extranigral regions and may account for the wide range of non-motor symptoms observed. The detailed mechanisms underlying the pathological changes in PD are not known but αSN is thought to play a central role. αSN is predominantly expressed in central nervous system neurons where it is localised to pre-synaptic terminals, regulates synaptic vesicle formation and neurotransmitter release [4,5] and can affect synaptic plasticity during learning [6]. However, recent evidence suggests that small oligomer aggregates of αSN accumulate at the pre-synaptic membrane and trigger synapse degeneration in PD and DLB [7-9]. The transfer of αSN to neighbouring neurons [10,11] may account for the stereotypical progression of αSN pathology through the brain similar to the staging of tau pathology in AD [12]. The loss of synapses in the hippocampus is characteristic of the PD patients that develop dementia [13] and in a rat model of α-synucleinopathy, synaptic degeneration preceded neuronal loss [14]. Thus, synapse degeneration is a common feature observed in PD, PDD and DLB.

There has been little study of the molecular mechanisms underpinning αSN induced synapse degeneration in these disorders. To investigate these mechanisms the effect of αSN on synapses in cultured cortical or hippocampal neurons was determined by quantifying the amount of synaptophysin using an enzyme-linked immunoassay (ELISA) [15]. Synaptophysin is a pre-synaptic membrane protein associated with recycling vesicles that are essential for neurotransmission [16,17] and the amount of synaptophysin has been used to access synaptic density in the brain [18-20] and cultured neurons [15]. An understanding of the molecular mechanisms that underlie αSN-induced synapse damage may help identify drugs that reduce this process.

## Results

### αSN causes synapse damage

**Figure 1:**
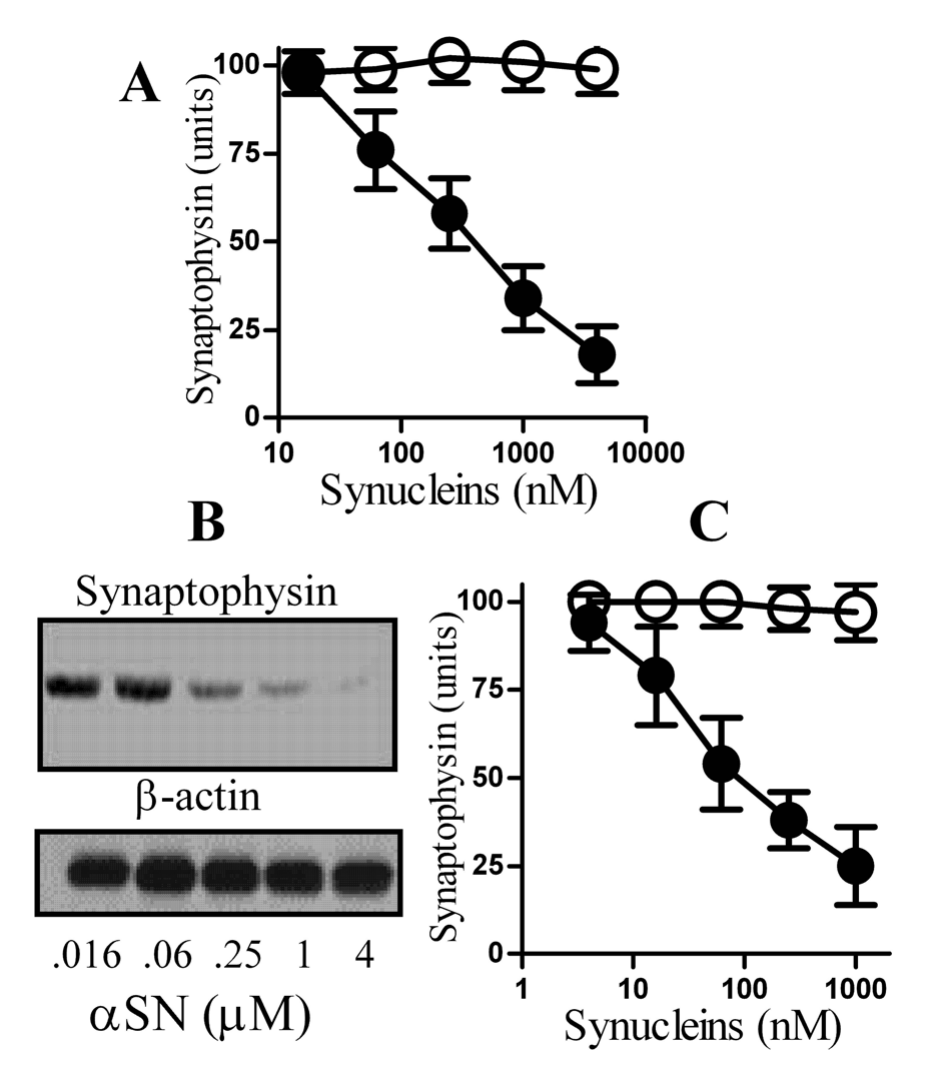
**αSN triggered the loss of synaptophysin from cultured neurons**. (A) The synaptophysin content of cortical neurons incubated for 24 hours with αSN (●) or βSN (○) as shown. Values shown are the mean amount of synaptophysin (units) ± SD, n = 15. (B) Immunoblots showing the amount of synaptophysin and β-actin in extracts from cortical neurons that had been incubated for 24 hours with αSN as shown. (C) The synaptophysin content of hippocampal neurons incubated for 24 hours with αSN (●) or βSN (○) as shown. Values shown are the mean amount of synaptophysin (units) ± SD, n = 12.

The synapse degeneration in PD and DLB that is associated with oligomers of αSN [7-9] was modelled *in vitro*. The addition of recombinant human αSN reduced the synaptophysin content of cortical neurons in a dose-dependent manner (Figure 1A). The synaptophysin content was reduced to 50% of control neurons (EC_50_) following the addition of 500 nM αSN. This effect of αSN on synapses occurred at concentrations that did not kill neurons; for example, the addition of 10 μM αSN reduced the synaptophysin content of cortical neurons by greater than 80% without affecting their viability as measured by thiazolyl blue tetrazolium (100% cell survival ± 8 compared with 96% ± 6, n = 9, P = 0.22). Similarly, immunoblots showed that αSN reduced the amount of synaptophysin in neuronal extracts without affecting the amount of β-actin (Figure 1B). The addition of βSN, another member of the synuclein family of proteins [6], did not affect the synaptophysin content of cortical neurons. We found that the addition of αSN, but not βSN, also reduced the amount of synaptophysin in hippocampal neurons (Figure 1C), an observation consistent with a report that the loss of synapses in the hippocampus is characteristic of the PD patients that develop dementia [13].

### αSN reduced synaptic vesicle recycling

**Figure 2:**
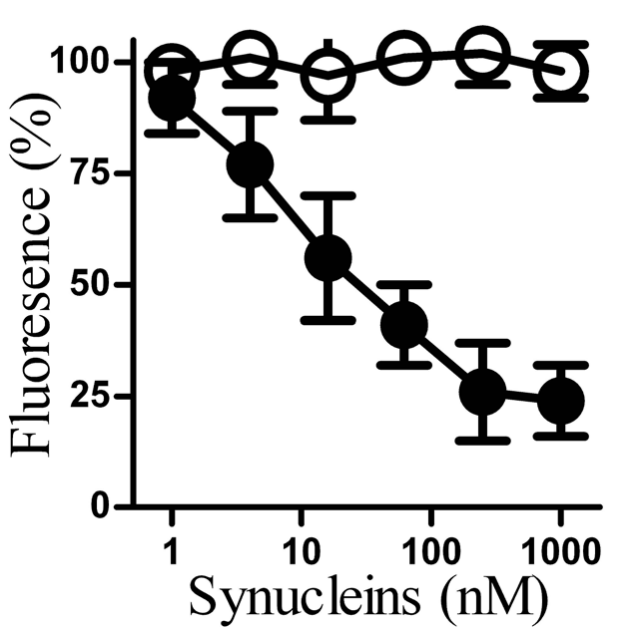
**αSN reduced synaptic vesicle recycling**. The amount of the fluorescent dye (FM1-43) taken up by synaptic vesicles in cortical neurons that had been incubated for 24 hours with different concentrations of αSN (●) or βSN (○) as shown and stimulated with ACh for 10 minutes. Values shown are the mean % fluorescence (where 100% = fluorescence in control cortical neurons) ± SD, n = 15.

The uptake of FM1-43, a fluorescent dye that is taken up into synaptic vesicles, was used as a measure of synaptic vesicle recycling and hence neurotransmission [21]. Here we report that the uptake of FM1-43 by cortical neurons was reduced following the addition of αSN, but not after the addition of βSN (Figure 2). This effect of αSN was observed at lower concentrations than that required to reduce the synaptophysin content of neurons and the concentration of αSN required to reduce synaptic vesicle recycling by 50% was approximately 30 nM.

### βSN reduced αSN-induced synapse damage

**Figure 3:**
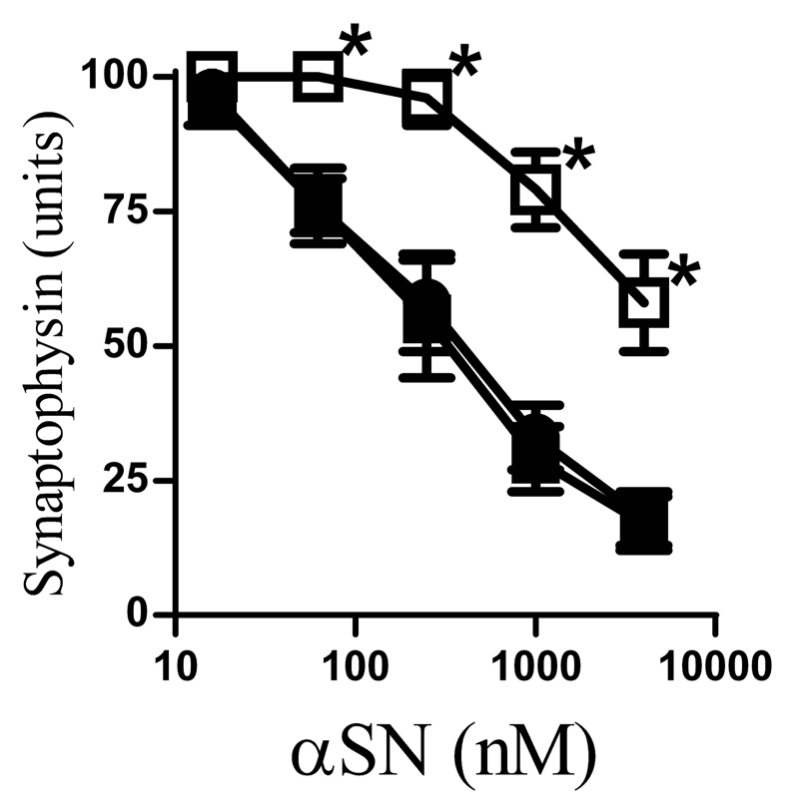
**βSN reduced the αSN-induced loss of synaptophysin**. The synaptophysin content of cortical neurons incubated with varying concentrations of αSN (●), αSN premixed with βSN (1:10)(□) or αSN premixed with human serum albumin (1:10)(■) for 24 hours. Values shown are the mean amount of synaptophysin (units) ± SD, n = 12. (*) = amount of synaptophysin significantly higher than in neurons incubated with the same concentration of αSN alone (P < 0.01).

Although transgenic mice studies showed that the expression of βSN reduced neurodegeneration in mice expressing human αSN [22,23], the molecular mechanisms underlying the interactions between αSN and βSN are unknown. We found that pre-mixing αSN with an excess of βSN (1:10) reduced the αSN-induced loss of synaptophysin in cortical neurons, whereas pre-mixing αSN with human serum albumin (1:10) had no affect (Figure 3). As this result may have been caused by a direct effect of βSN on neurons, cortical neurons were pre-treated with 10 μM βSN, washed and then incubated with αSN. Pre-treatment of neurons with βSN did not affect the loss of synaptophysin induced by αSN (data not shown).

### Aβ_1-42 _enhanced αSN-induced synapse damage

**Figure 4:**
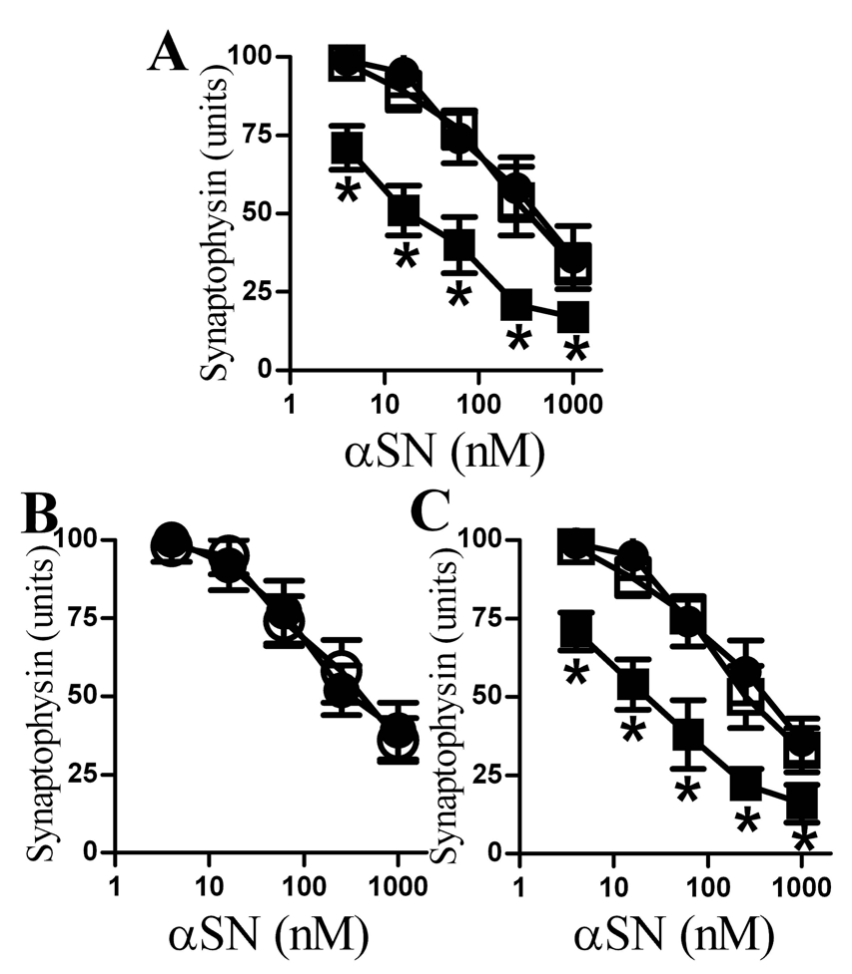
**Aβ_1-42 _enhanced the αSN-induced loss of synaptophysin**. (A) The synaptophysin content of cortical neurons incubated for 24 hours with varying concentrations of αSN (●), αSN premixed with Aβ_1-42 _(50:1) (■) or αSN premixed with Aβ_42-1 _(50:1) (□). Values shown are the mean amount of synaptophysin (units) ± SD, n = 18. (*) = amount of synaptophysin significantly lower than in neurons incubated with the same concentration of αSN alone (P < 0.01). (B) The synaptophysin content of cortical neurons pre-treated for 1 hour with control medium (●) or 1 nM Aβ_1-42 _(○) and incubated for 24 hours with αSN as shown. Values shown are the mean amount of synaptophysin (units) ± SD, n = 12. (C) The synaptophysin content of cortical neurons incubated for 24 hours with αSN (●), αSN premixed with 7PA2-CM (■) or αSN premixed with CHO-CM (□). Values shown are the mean amount of synaptophysin (units) ± SD, n = 12. (*) = amount of synaptophysin significantly lower than in neurons incubated with the same concentration of αSN alone (P < 0.01).

The amyloid hypothesis of Alzheimer's disease (AD) pathogenesis maintains that the primary event is the production of neurotoxic amyloid-β (Aβ) peptides following the proteolytic cleavage of the amyloid precursor protein into different sized fragments [24,25]. These fragments include Aβ_1-42 _which is widely regarded as a major pathogenic species in AD [26]. Since recent reports showed that αSN and Aβ_1-42 _co-exist in heterologous oligomers [27,28] the effect of Aβ_1-42 _on αSN-induced loss of synaptophysin was examined by pre-mixing the two peptides. The addition of Aβ_1-42 _in the ratio (1:50) increased αSN-induced synapse damage (Figure 4A). Thus, while the EC_50 _of αSN alone was 500 nM, the EC_50 _of Aβ_1-42_:αSN (1:50) was 25 nM. These concentrations of Aβ_1-42 _did not affect synapses when added on their own. In contrast, pre-mixing αSN with the control peptide Aβ_42-1 _(1:50) did not affect αSN-induced loss of synaptophysin. Since the predominant Aβ species found within the brain is Aβ_1-40 _[29] the effect of Aβ_1-40 _on αSN was also tested. However, there was no significant difference in the synaptophysin content of cortical neurons incubated with αSN and neurons incubated with Aβ_1-40_/αSN (1:50) (data not shown). These results may have been caused by a direct effect of Aβ_1-42 _on the neurons. Our observation that pre-treatment of cortical neurons with 1 nM Aβ_1-42 _did not affect αSN-induced loss of synaptophysin (Figure 4B) suggested that Aβ_1-42 _did not sensitise neurons to the effects of αSN.

The ability of synthetic Aβ peptides to self associate results in a mixture of physical complexes ranging from small soluble oligomers to large fibrils. Since the dynamic nature of Aβ aggregation means that it is difficult to ascribe biological function to specific Aβ assemblies using synthetic peptides, the activity of naturally derived, stable Aβ oligomers was also examined. We found that pre-mixing αSN with 7PA2-conditioned medium (7PA2-CM), which contained naturally secreted stable Aβ oligomers [30,31], increased the αSN-induced loss of synaptophysin in cortical neurons (Figure 4C). In contrast, pre-mixing αSN with CHO-CM had no effect.

### Aβ enhanced αSN-induced inhibition of synaptic vesicle recycling

**Figure 5:**
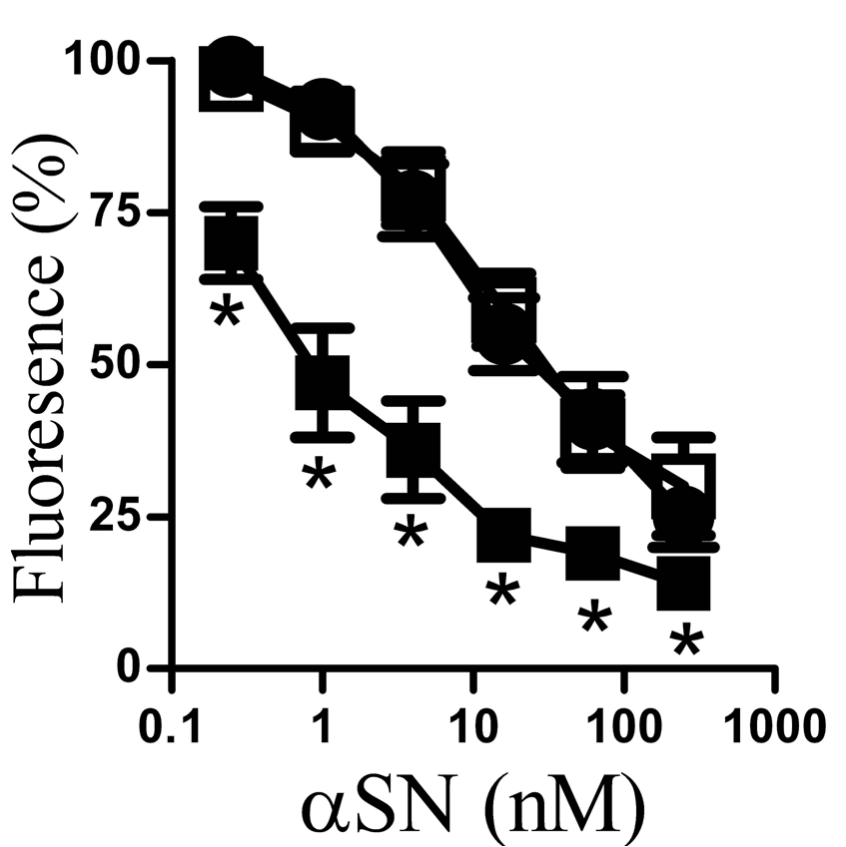
**Aβ_1-42 _enhanced the αSN-induced inhibition of synaptic vesicle recycling**. The amount of the fluorescent dye (FM1-43) taken up into synaptic vesicles in cortical neurons that had been incubated for 24 hours with different concentrations of αSN (●), or with αSN that had been premixed with either 7PA2-CM (■) or with CHO-CM (□) as shown and stimulated with ACh for 10 minutes. Values shown are the mean % fluorescence (where 100% = fluorescence in vehicle treated cortical neurons) ± SD, n = 18. (*) = Fluorescence significantly lower than in neurons incubated with the same concentration of αSN alone (P < 0.01).

The addition of Aβ oligomers also affected αSN-induced inhibition of synaptic vesicle recycling. Thus, pre-mixing αSN with 7PA2-CM enhanced αSN-induced inhibition of FM1-43 uptake into synapses. The concentration of αSN alone required to reduce synaptic vesicle recycling by 50% was 30 nM, while the concentration of αSN that had been mixed with 7PA2-CM to have a similar level of effect was 1 nM (Figure 5).

### αSN enhanced Aβ_1-42_-induced synapse damage

**Figure 6:**
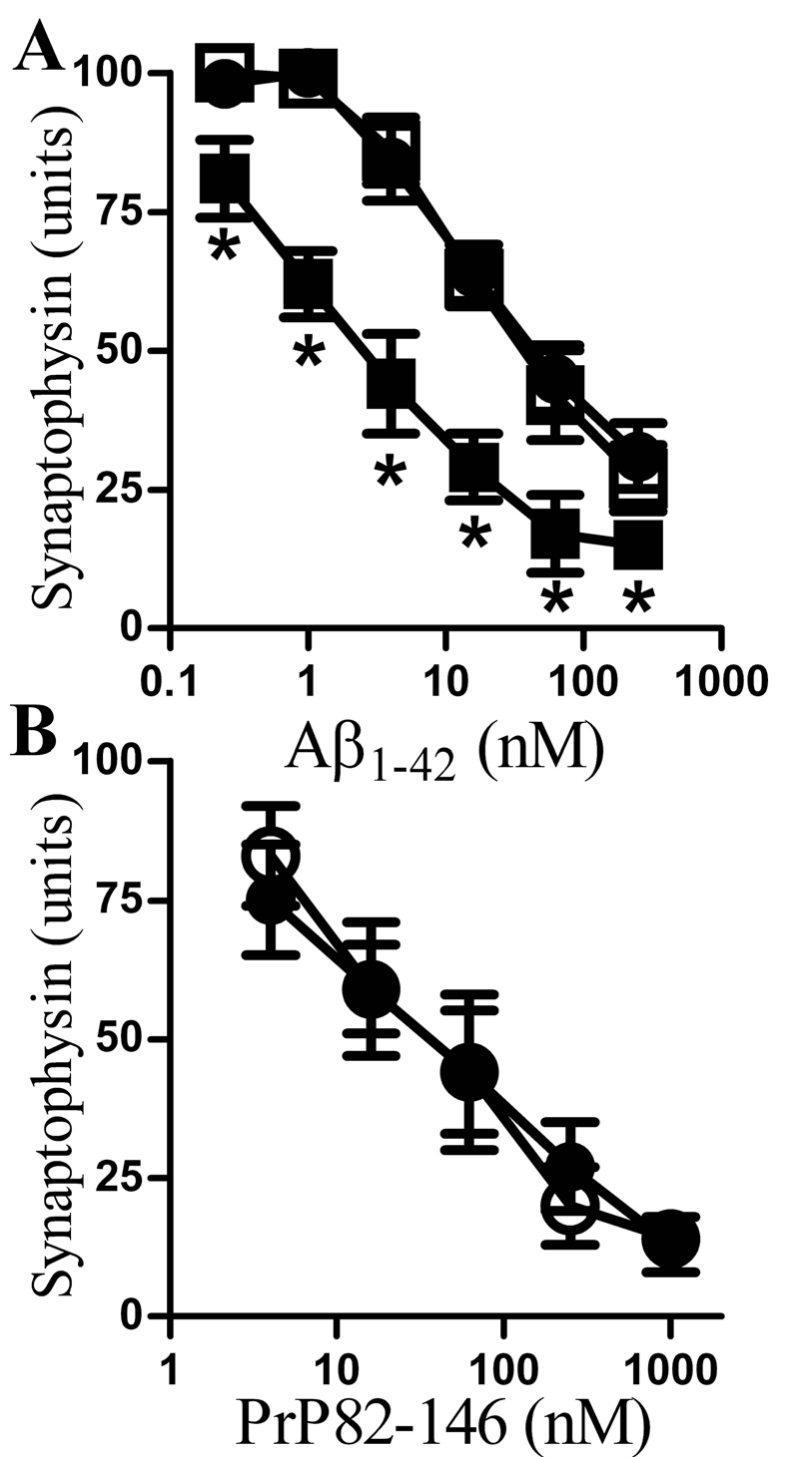
**αSN enhanced the Aβ_1-42_-induced loss of synaptophysin**. (A) The synaptophysin content of cortical neurons incubated for 24 hours with Aβ_1-42 _(●) Aβ_1-42 _premixed with αSN (1:2) (■) or Aβ_1-42 _premixed with βSN (1:2) (□). Values shown are the mean amount of synaptophysin (units) ± SD, n = 15. (*) = amount of synaptophysin significantly lower than in neurons incubated with the same concentration of Aβ_1-42 _alone (P < 0.01). (B) The synaptophysin content of cortical neurons incubated for 24 hours with PrP82-146 (●) or PrP82-146 that had been premixed with αSN (1:5) (○). Values shown are the mean amount of synaptophysin (units) ± SD, n = 15.

Next we examined whether non-toxic concentrations of αSN affected Aβ_1-42_-induced loss of synaptophysin. Here we show that pre-mixing Aβ_1-42 _with αSN increased the Aβ_1-42_-induced loss of synaptophysin from neurons (Figure 6). Thus, while the EC_50 _of Aβ_1-42 _alone was 50 nM, the EC_50 _of αSN:Aβ_1-42 _(2:1) was 5 nM. In contrast, the addition of βSN (2:1) did not affect Aβ_1-42_-induced loss of synaptophysin. Pre-treatment of cortical neurons with 10 nM αSN did not affect Aβ_1-42_-induced loss of synaptophysin (data not shown). We also sought to determine if αSN affected another peptide that caused synapse damage. Synapse degeneration is a feature of human and experimental prion diseases [32,33] which was modelled by the addition of the prion-derived peptide PrP82-146 to cortical neurons [15]. As shown in Figure 6B, there was no difference in the synaptophysin content of cortical neurons incubated with PrP82-146 and those incubated with a combination of αSN/PrP82-146 (2:1).

### αSN did not affect the accumulation of Aβ_1-42 _in synapses

**Figure 7:**
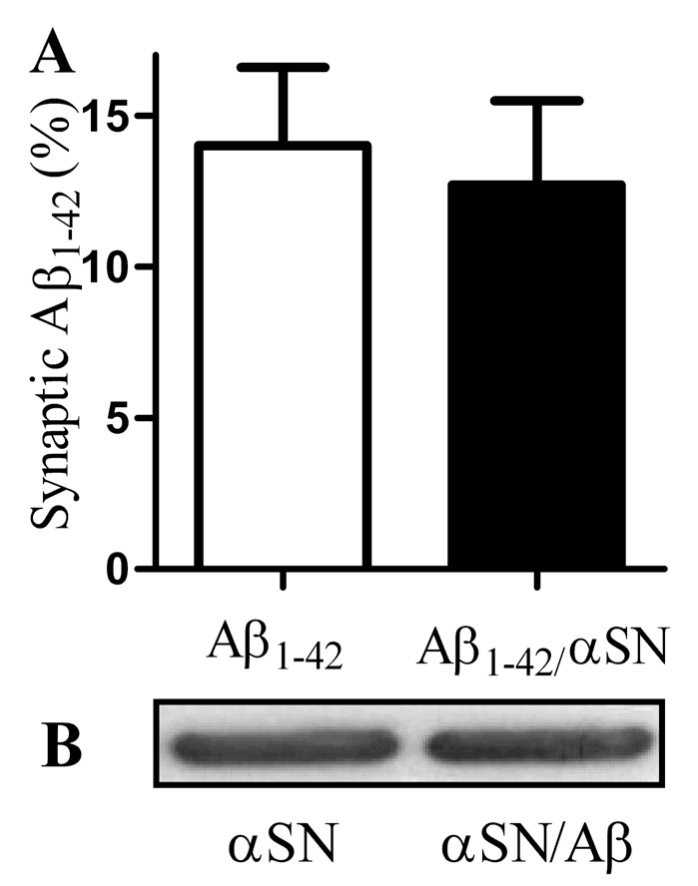
**αSN did not affect the accumulation of Aβ_1-42 _in synapses**. (A) The amount of biotinylated-Aβ_1-42 _found in synaptosomes derived from cortical neurons incubated for 1 hour with 100 nM Aβ_1-42 _(□) or 100 nM Aβ_1-42 _that had been pre-mixed with 500 nM αSN (■). Values shown are the mean amount of Aβ_1-42 _expressed as a % of the amount added ± SD, n = 12. (B) Immunoblot showing the amount of αSN in synaptosomes collected from cortical neurons incubated with 200 nM αSN alone or with 200 nM αSN pre-mixed with Aβ_1-42 _(50:1).

We explored the possibility that αSN increased the binding of Aβ_1-42 _to synapses as an explanation of the effect of αSN on Aβ_1-42_-induced loss of synaptophysin. Time course studies showed that Aβ_1-42 _accumulated in synaptosomes isolated from cortical neurons after 1 hour. Therefore cortical neurons were incubated with 100 nM biotinylated Aβ_1-42_, or 100 nM biotinylated-Aβ_1-42 _that had been pre-mixed with 500 nM αSN for 1 hour. The amount of biotinylated Aβ_1-42 _found in synaptosomes isolated from these neurons was not altered by pre-mixing with αSN (Figure 7A), indicating that αSN did not alter the binding, or trafficking of Aβ_1-42 _to synapses. The effect of Aβ_1-42 _on the accumulation of αSN in synapses was also examined. Cortical neurons were incubated with 200 nM αSN, or a combination of 4 nM Aβ_1-42 _and 200 nM αSN (1:50), for 1 hour and synaptosomes prepared. Immunoblots showed that the amount of αSN found within synapses was not affected by presence of Aβ_1-42 _(Figure 7B).

## Discussion

The loss of synapses is a prominent feature of many neurodegenerative diseases including AD, PDD and LBD. The main mediators of neuropathology in PDD and LBD are thought to be oligomers of αSN [7,9] and in this study the addition of αSN impaired synapse function and triggered a loss of synaptophysin from cortical neurons. These effects occurred at concentrations of αSN that did not affect neuronal survival; an observation consistent with reports that synapse degeneration preceded neuronal loss in a rat model of α-synucleinopathy [14]. A reduction in the synaptophysin content of hippocampal neurons was observed after incubation with αSN, consistent with reports that a loss of synapses in the hippocampus is characteristic of the PD patients that develop dementia [13].

The addition of βSN did not affect synapses indicating that synaptic defects were dependent upon the specific amino acid sequence of αSN. Recent reports from a transgenic mouse model of PD showed that the expression of βSN reduced the accumulation of αSN and neurodegeneration in mice expressing human αSN [22,23]. Another study showed that βSN formed mixed oligomers with αSN [34]. In this study we showed that mixing with βSN reduced the loss of synaptophysin induced by αSN; results consistent with the idea that molecular interactions between αSN and βSN affect the toxicity of αSN.

Approximately 25% of AD patients develop parkinsonism and 50% of PD patients develop AD-type dementia after 65 years of age [35]. In addition, 70% of patients with sporadic AD display αSN-positive, LB-like inclusions in the amygdala and limbic structures [36-38]. The loss of synapses that occurs in AD is associated with the production of Aβ oligomers [39-41]. Both Aβ and αSN accumulate in the brain in DLB [3] and levels of αSN are increased in AD [42], observations which suggest that interactions between αSN and Aβ affect the pathogenesis of AD, PDD and DLB [43].

The addition of small amounts of Aβ_1-42_, which had no effect on synapses on their own, increased the effects of αSN on synapses. Critically pre-treatment with Aβ_1-42 _did not sensitize cortical neurons to the synaptic effects of αSN and while we cannot exclude the possibility of a transient sensitizing effect of Aβ_1-42_, our results suggest that direct interactions between Aβ_1-42 _and αSN increased the toxicity of αSN. The studies using synthetic Aβ_1-42 _peptides were complimented by studies using 7PA2-CM containing naturally secreted, stable Aβ oligomers [30,31]. Pre-mixing with 7PA2-CM also increased the effects of αSN upon synaptic vesicle recycling and synapse damage. Relatively small amounts of Aβ_1-42 _(1:50) were required to facilitate the αSN-induced loss of synaptophysin suggesting that Aβ_1-42 _seeded the formation of toxic αSN oligomers; an observation consistent with reports that Aβ promotes the aggregation of αSN in transgenic mice [44]. Conversely, the addition of non-toxic concentrations of αSN increased Aβ_1-42_-induced loss of synaptophysin.

Aβ_1-42 _exists in multiple forms from small soluble toxic oligomers to large insoluble amyloid fibrils. As the toxicity of Aβ_1-42 _is affected by its state of aggregation [45,46] the addition of αSN may stabilize Aβ_1-42 _oligomers in a toxic configuration. Mixing αSN with Aβ_1-42 _did not increase the amount of αSN or Aβ_1-42 _found within synapses showing that the increased toxicity of hetero-oligomers was not due to increased binding of Aβ_1-42 _or αSN to synapses.

Although Aβ_1-42 _is considered to be the major neurotoxin generated in AD [47-50] other Aβ fragments are produced [24,25,51] and since Aβ_1-40 _is the predominant Aβ species formed in AD [29,52] the effect of Aβ_1-40 _on αSN-induced loss of synaptophysin was also tested. We found that αSN did not affect the reduction in synaptophysin in response to Aβ_1-40_, nor did Aβ_1-40 _affect the reduction in synaptophysin induced by αSN. Synapse degeneration is also a feature of human and experimental prion diseases [32,33] and the prion-derived peptide PrP82-146 triggered a reduction in synaptophysin in cortical neurons [15]. Although PrP82-146 has similar biophysical properties to Aβ_1-42 _in that it adopts a β-helix-rich conformation, forms oligomers and fibrils which are protease resistant [53], αSN did not affect PrP82-146-induced loss of synaptophysin.

## Conclusions

We conclude that the addition of αSN reduced the synaptophysin content of cultured cortical and hippocampal neurons, a model that mimics the synapse damage observed in PDD and DLB. The effect of αSN was modified by other proteins found in the central nervous system including βSN which reduced the effects of αSN, and Aβ_1-42 _which increased the effects of αSN on synapses. Conversely, αSN increased the effects of Aβ_1-42 _on synapses. Our results suggest that interactions between the synucleins and Aβ peptides may affect synapses in AD, PDD and Lewy body disorders.

## Methods

### Primary neuronal cultures

Cortical neurons were prepared from the brains of mouse embryos (day 15.5) as described [15]. Neurons were plated at 2 × 10^5 ^cells/well in pre-coated 48 well plates (5 μg/ml poly-L-lysine) in Ham's F12 (PAA) containing 5% foetal calf serum (FCS) for 2 hours. Cultures were shaken (600 r.p.m for 5 minutes) and non-adherent cells removed by 3 washes in PBS. Neurons were grown in neurobasal medium (NBM) containing B27 components (PAA) for 10 days. Immunohistochemistry showed that the cells were greater than 97% neurofilament positive. Fewer than 3% of cells stained for glial fibrillary acidic protein (astrocytes) or for F4/80 (microglial cells). Hippocampal neurons were prepared from the brains of adult mice as described [54]. Hippocampi were dissected from brains and triturated in Ham's F12 containing 5% FCS, 0.35% glucose, 0.025% trypsin, and 0.1% type IV collagenase. After 30 minutes at 37°C, the cells were triturated and the cell suspension was passed through a 100 μM cell strainer. Cells were collected, washed twice and plated at 2 × 10^5 ^cells/well in 48 well plates (pre-coated with poly-L-lysine). After 24 hours cultures were shaken (600 r.p.m for 5 minutes) to remove non-adherent cells, washed twice and the remaining neurons were cultured in NBM/B27 and 10 ng/ml glial-derived neurotrophic factor (Sigma) for 7 days. Neurons were incubated with peptides for 24 hours and the amount of synaptophysin in cell extracts measured.

### Cell extracts

Neurons were washed 3 times with PBS and homogenised in a buffer containing 150 mM NaCl, 10 mM Tris-HCl, pH 7.4, 10 mM EDTA, 0.5% Nonidet P-40, 0.5% sodium deoxycholate, 0.2% sodium dodecyl sulphate (SDS) and mixed protease inhibitors (4-(2-Aminoethyl) benzenesulfonyl fluoride hydrochloride (AEBSF), Aprotinin, Leupeptin, Bestain, Pepstatin A and E-46) (Sigma) at 10^6 ^cells/ml. For immunoblots cells were homogenised in extraction buffer (as above) at 10^7 ^cells/ml and digested with DNAse (Sigma) for 1 hour at 37°C. Cell debris was removed by low speed centrifugation (300 × *g *for 5 minutes).

### Synaptophysin ELISA

The amount of **s**ynaptophysin in neuronal extracts was measured by ELISA [15]. Briefly, the capture mAb was anti-synaptophysin MAB368 (Millipore). Samples were added for 1 hour and bound synaptophysin was detected using rabbit polyclonal anti-synaptophysin (Abcam) followed by a biotinylated anti-rabbit IgG (Dako), extravidin-alkaline phosphatase and 1 mg/ml 4-nitrophenol phosphate. Absorbance was measured on a microplate reader at 405 nm and the synaptophysin content of samples was expressed as units where 100 units was defined as the amount of synaptophysin in untreated neurons.

### Synaptic vesicle recycling

The fluorescent styryl dye FM1-43 (Biotium) that is readily taken up into synaptic recycling vesicles [55] was used to determine synaptic activity as described [21]. Treated neurons were incubated with 1 μg/ml FM1-43 and 1 μM acetylcholine (ACh) for 10 minutes, washed 5 times in ice cold PBS and solubilised in methanol at 1 × 10^6 ^neurons/ml. Soluble extracts were transferred into Sterlin 96 well black microplates and fluorescence was measured using excitation at 480 nm and emission at 625 nm. Background fluorescence was subtracted and samples were expressed as "% fluorescence" where 100% fluorescence was defined as the amount of fluorescence in untreated neurons incubated with FM1-43 and ACh.

### Synaptosome preparations

Synaptosomes were prepared on a discontinuous Percoll gradient [56]. Briefly, 10^6 ^cortical neurons were homogenized at 4°C in 1 ml of SED solution (0.32 M sucrose, 50 mM Tris-HCl, pH 7.2, 1 mM EDTA, and 1 mM dithiothreitol, and centrifuged at 1000 × *g *for 10 minutes. The supernatant was transferred to a gradient of 3, 7, 15, and 23% Percoll in SED solution and centrifuged at 16,000 × *g *for 30 minutes at 4°C. The synaptosomes were collected from the interface of the 15% and 23% Percoll steps. The fraction was washed twice (16,000 × *g *for 30 minutes at 4°C) and suspended in extraction buffer containing 150 mM NaCl, 10 mM Tris-HCl, pH 7.4, 10 mM EDTA, 0.2% sodium dodecyl sulphate and mixed protease inhibitors.

### Biotinylated Aβ_1-42 _ELISA

The amounts of biotinylated Aβ_1-42 _in extracts were determined by ELISA. Nunc Maxisorb Immunoplates were coated with 1 μg/ml protein A (Innova) followed by 0.1 μg/ml mAb reactive to amino acids 1 to 16 of β-amyloid (clone 6E10 - Signet) and blocked with 5% milk powder. Samples were boiled in 0.2% SDS, cooled and incubated for 1 hour. Biotinylated Aβ_1-42 _was detected with extravidin-alkaline phosphatase and 1 mg/ml 4-nitrophenyl phosphate (Sigma). Absorbance was measured on a microplate reader at 405 nm and results were calculated by reference to a standard curve generated form serial dilutions of biotinylated Aβ_1-42_.

### Western analysis

Samples were mixed with an equal volume of Laemmli buffer, boiled, and subjected to electrophoresis on a 15% polyacrylamide gel (Invitrogen). Proteins were transferred onto a Hybond-P PVDF membrane (Amersham Biotech) by semi-dry blotting. Membranes were blocked using 10% milk powder; synaptophysin was detected using a mouse monoclonal antibody (mAb) anti-synaptophysin SY38 (Abcam), β-actin was detected by incubation with a mouse mAb (clone AC-74, Sigma) and human αSN was detected by incubation with mAb 211 raised against amino acids 121 to 125 of human αSN (Santa Cruz Biotech). These were visualised using a combination of biotinylated secondary antibodies (Dako), extravidin-peroxidase and an enhanced chemiluminescence kit.

### Peptides

Recombinant human αSN and βSN were purchased from Sigma. Synthetic peptides containing the amino acids 1 to 42 (Aβ_1-42_) or 1 to 40 (Aβ_1-40_) of the Aβ protein, biotinylated-Aβ_1-42 _and a control peptide consisting of amino acids 1 to 42 in reverse order (Aβ_42-1_) were obtained from Bachem. Peptides containing amino acids 82 to 146 of the human PrP protein (PrP82-146) and was a gift from Professor Salmona (Mario Negri, Milan). Aβ peptides were first dissolved in hexafluoroisopropanol, lyophilised and subsequently solubilised and stored at 1 mM in DMSO. Stock solutions of peptides were stored at 1 mM, thawed on the day of use and diluted/mixed in NBM for 1 hour at 37°C. Dilutions/mixtures were subjected to vigorous shaking (Disruptor Genie, full power for 10 minutes) before they were added to neurons. Chinese hamster ovary (CHO) cells stably transfected with a cDNA encoding APP_751 _containing the Val717Phe familial AD mutation (referred to as 7PA2 cells) were cultured in DMEM with 10% FCS [30,31]. Conditioned medium from these cells contains stable Aβ oligomers (7PA2-CM). Conditioned medium from non-transfected CHO cells (CHO-CM) was used as controls. These were mixed with peptides and subjected to vigorous shaking (as above), diluted in NBM and added to neurons.

### Statistical Analysis

Differences between treatment groups were determined by 2 sample, paired T-tests. For all statistical tests significance was set at the 1% level.

## Abbreviations

(ACh): Acetylcholine; (AD): Alzheimer's disease; (αSN): alpha-synuclein; (βSN): beta-synuclein; (Aβ): amyloid-β; (CHO): Chinese hamster ovary; (CM): conditioned medium; (DLB): dementia with Lewy bodies; (DMSO): di-methyl sulphoxide; (ELISA): Enzyme linked immunoassay; (NBM): neurobasal medium; (LB): Lewy body; (PD): Parkinson's disease; (PDD): Parkinson's diseases dementia; (PBS): phosphate buffered saline.

## Competing interests

The authors declare that they have no competing interests.

## Authors' contributions

CB was responsible for the conception, planning and performance of experiments and for writing the manuscript. AW and SG contributed to the planning of experiments, interpretation of results and the writing of the manuscript. All authors approved the final manuscript.

## References

[B1] Poewe W Non-motor symptoms in Parkinson's disease Eur J Neurol 2008 15 Suppl 1 14 20 10.1111/j.1468-1331.2008.02056.x 18353132

[B2] Aarsland D Andersen K Larsen JP Lolk A Kragh-Sorensen P Prevalence and characteristics of dementia in Parkinson disease: an 8-year prospective study Arch Neurol 2003 60 3 387 392 10.1001/archneur.60.3.387 12633150

[B3] McKeith IG Dickson DW Lowe J Emre M O'Brien JT Feldman H Cummings J Duda JE Lippa C Perry EK Diagnosis and management of dementia with Lewy bodies: third report of the DLB Consortium Neurology 2005 65 12 1863 1872 10.1212/01.wnl.0000187889.17253.b1 16237129

[B4] Bonini NM Giasson BI Snaring the function of alpha-synuclein Cell 2005 123 3 359 361 10.1016/j.cell.2005.10.017 16269324

[B5] Cabin DE Shimazu K Murphy D Cole NB Gottschalk W McIlwain KL Orrison B Chen A Ellis CE Paylor R Synaptic vesicle depletion correlates with attenuated synaptic responses to prolonged repetitive stimulation in mice lacking alpha-synuclein J Neurosci 2002 22 20 8797 8807 12388586 10.1523/JNEUROSCI.22-20-08797.2002 PMC6757677

[B6] Clayton DF George JM The synucleins: a family of proteins involved in synaptic function, plasticity, neurodegeneration and disease Trends Neurosci 1998 21 6 249 254 10.1016/S0166-2236(97)01213-7 9641537

[B7] Kazantsev AG Kolchinsky AM Central role of alpha-synuclein oligomers in neurodegeneration in Parkinson disease Arch Neurol 2008 65 12 1577 1581 10.1001/archneur.65.12.1577 19064744

[B8] Kramer ML Schulz-Schaeffer WJ Presynaptic α-Synuclein Aggregates, Not Lewy Bodies, Cause Neurodegeneration in Dementia with Lewy Bodies J Neurosci 2007 27 6 1405 1410 10.1523/JNEUROSCI.4564-06.2007 17287515 PMC6673583

[B9] Lee VMY Trojanowski JQ Mechanisms of Parkinson's Disease Linked to Pathological [alpha]-Synuclein: New Targets for Drug Discovery Neuron 2006 52 1 33 38 10.1016/j.neuron.2006.09.026 17015225

[B10] Desplats P Lee H-J Bae E-J Patrick C Rockenstein E Crews L Spencer B Masliah E Lee S-J Inclusion formation and neuronal cell death through neuron-to-neuron transmission of α-synuclein Proc Natl Acad Sci USA 2009 106 31 13010 13015 10.1073/pnas.0903691106 19651612 PMC2722313

[B11] Brundin P Melki R Kopito R Prion-like transmission of protein aggregates in neurodegenerative diseases Nat Rev Mol Cell Biol 2010 11 4 301 307 10.1038/nrm2873 20308987 PMC2892479

[B12] Braak H Del Tredici K Rub U de Vos RA Jansen Steur EN Braak E Staging of brain pathology related to sporadic Parkinson's disease Neurobiol Aging 2003 24 2 197 211 10.1016/S0197-4580(02)00065-9 12498954

[B13] Galvin JE Uryu K Lee VM Trojanowski JQ Axon pathology in Parkinson's disease and Lewy body dementia hippocampus contains alpha-, beta-, and gamma-synuclein Proc Natl Acad Sci USA 1999 96 23 13450 13455 10.1073/pnas.96.23.13450 10557341 PMC23968

[B14] Chung CY Koprich JB Siddiqi H Isacson O Dynamic changes in presynaptic and axonal transport proteins combined with striatal neuroinflammation precede dopaminergic neuronal loss in a rat model of AAV alpha-synucleinopathy J Neurosci 2009 29 11 3365 3373 10.1523/JNEUROSCI.5427-08.2009 19295143 PMC2693917

[B15] Bate C Tayebi M Salmona M Diomede L Williams A Polyunsaturated fatty acids protect against prion-mediated synapse damage in vitro Neurotox Res 2010 17 3 203 214 10.1007/s12640-009-9093-2 19644728

[B16] Elferink LA Scheller RH Synaptic vesicle proteins and regulated exocytosis J Cell Sci 1993 17 75 79 10.1242/jcs.1993.supplement_17.11 8144706

[B17] Daly C Sugimori M Moreira JE Ziff EB Llinas R Synaptophysin regulates clathrin-independent endocytosis of synaptic vesicles Proc Natl Acad Sci USA 2000 97 11 6120 6125 10.1073/pnas.97.11.6120 10823955 PMC18568

[B18] Reddy PH Mani G Park BS Jacques J Murdoch G Whetsell W Jr Kaye J Manczak M Differential loss of synaptic proteins in Alzheimer's disease: implications for synaptic dysfunction J Alzheimers Dis 2005 7 2 103 117 15851848 10.3233/jad-2005-7203

[B19] Counts SE Nadeem M Lad SP Wuu J Mufson EJ Differential expression of synaptic proteins in the frontal and temporal cortex of elderly subjects with mild cognitive impairment J Neuropath Exp Neurol 2006 65 6 592 601 10.1097/00005072-200606000-00007 16783169

[B20] Masliah E Terry RD Alford M DeTeresa R Hansen LA Cortical and subcortical patterns of synaptophysinlike immunoreactivity in Alzheimer's disease Am J Path 1991 138 1 235 246 1899001 PMC1886043

[B21] Bate C Williams A Amyloid-β_1-40 _Inhibits Amyloid-β_1-42 _Induced Activation of Cytoplasmic Phospholipase A_2 _and Synapse Degeneration J Alzheimers Dis 2010 21 3 985 993 20634578 10.3233/JAD-2010-100528

[B22] Hashimoto M Rockenstein E Mante M Mallory M Masliah E β-Synuclein inhibits alpha-synuclein aggregation: a possible role as an anti-parkinsonian factor Neuron 2001 32 2 213 223 10.1016/S0896-6273(01)00462-7 11683992

[B23] Park JY Lansbury PT Jr Beta-synuclein inhibits formation of alpha-synuclein protofibrils: a possible therapeutic strategy against Parkinson's disease Biochemistry 2003 42 13 3696 3700 10.1021/bi020604a 12667059

[B24] Vassar R Citron M Aβ-Generating Enzymes: Recent Advances in β and γ-Secretase Research Neuron 2000 27 3 419 422 10.1016/S0896-6273(00)00051-9 11055423

[B25] Hardy J Selkoe DJ The amyloid hypothesis of Alzheimer's disease: progress and problems on the road to therapeutics Science 2002 297 5580 353 356 10.1126/science.1072994 12130773

[B26] Yankner BA Lu T Amyloid beta-protein toxicity and the pathogenesis of Alzheimer disease JBiolChem 2009 284 8 4755 4759 10.1074/jbc.R800018200 PMC2643502 18957434

[B27] Tsigelny IF Crews L Desplats P Shaked GM Sharikov Y Mizuno H Spencer B Rockenstein E Trejo M Platoshyn O Mechanisms of Hybrid Oligomer Formation in the Pathogenesis of Combined Alzheimer's and Parkinson's Diseases PLoS ONE 2008 3 9 e3135 10.1371/journal.pone.0003135 18769546 PMC2519786

[B28] Mandal P Pettegrew J Masliah E Hamilton R Mandal R Interaction between Aβ Peptide and α Synuclein: Molecular Mechanisms in Overlapping Pathology of Alzheimer's and Parkinson's in Dementia with Lewy Body Disease Neurochem Res 2006 31 9 1153 1162 10.1007/s11064-006-9140-9 16947080

[B29] Borchelt DR Thinakaran G Eckman CB Lee MK Davenport F Ratovitsky T Prada CM Kim G Seekins S Yager D Familial Alzheimer's disease-linked presenilin 1 variants elevate Aβ_1-42/1-40 _ratio in vitro and in vivo Neuron 1996 17 5 1005 1013 10.1016/S0896-6273(00)80230-5 8938131

[B30] Koo EH Squazzo SL Evidence that production and release of amyloid β-protein involves the endocytic pathway JBiolChem 1994 269 26 17386 17389 8021238

[B31] Podlisny MB Ostaszewski BL Squazzo SL Koo EH Rydell RE Teplow DB Selkoe DJ Aggregation of secreted amyloid β-protein into sodium dodecyl sulfate-stable oligomers in cell culture JBiolChem 1995 270 16 9564 9570 10.1074/jbc.270.16.9564 7721886

[B32] Jeffrey M Halliday WG Bell J Johnston AR MacLeod NK Ingham C Sayers AR Brown DA Fraser JR Synapse loss associated with abnormal PrP precedes neuronal degeneration in the scrapie-infected murine hippocampus Neuropath Appl Neurobiol 2000 26 1 41 54 10.1046/j.1365-2990.2000.00216.x 10736066

[B33] Ferrer I Synaptic pathology and cell death in the cerebellum in Creutzfeldt-Jakob disease Cerebellum 2002 1 3 213 222 10.1080/14734220260418448 12879983

[B34] Israeli E Sharon R beta-Synuclein occurs in vivo in lipid-associated oligomers and forms hetero-oligomers with alpha-synuclein J Neurochem 2009 108 2 465 474 10.1111/j.1471-4159.2008.05776.x 19012742 PMC2832289

[B35] Hansen L Salmon D Galasko D Masliah E Katzman R DeTeresa R Thal L Pay MM Hofstetter R Klauber M The Lewy body variant of Alzheimer's disease: a clinical and pathologic entity Neurology 1990 40 1 1 8 2153271 10.1212/wnl.40.1.1

[B36] Lippa CF Fujiwara H Mann DM Giasson B Baba M Schmidt ML Nee LE O'Connell B Pollen DA St George-Hyslop P Lewy bodies contain altered alpha-synuclein in brains of many familial Alzheimer's disease patients with mutations in presenilin and amyloid precursor protein genes Am J Pathol 1998 153 5 1365 1370 9811326 10.1016/s0002-9440(10)65722-7 PMC1853391

[B37] Trojanowski JQ Goedert M Iwatsubo T Lee VM Fatal attractions: abnormal protein aggregation and neuron death in Parkinson's disease and Lewy body dementia Cell Death Differ 1998 5 10 832 837 10.1038/sj.cdd.4400432 10203692

[B38] Hamilton RL Lewy bodies in Alzheimer's disease: a neuropathological review of 145 cases using alpha-synuclein immunohistochemistry Brain Pathol 2000 10 3 378 384 10.1111/j.1750-3639.2000.tb00269.x 10885656 PMC8098522

[B39] Shankar GM Li S Mehta TH Garcia-Munoz A Shepardson NE Smith I Brett FM Farrell MA Rowan MJ Lemere CA Amyloid-β protein dimers isolated directly from Alzheimer's brains impair synaptic plasticity and memory Nat Med 2008 14 8 837 842 10.1038/nm1782 18568035 PMC2772133

[B40] Takahashi RH Almeida CG Kearney PF Yu F Lin MT Milner TA Gouras GK Oligomerization of Alzheimer's β-Amyloid within Processes and Synapses of Cultured Neurons and Brain J Neurosci 2004 24 14 3592 3599 10.1523/JNEUROSCI.5167-03.2004 15071107 PMC6729733

[B41] Walsh DM Klyubin I Fadeeva JV Cullen WK Anwyl R Wolfe MS Rowan MJ Selkoe DJ Naturally secreted oligomers of amyloid β protein potently inhibit hippocampal long-term potentiation in vivo Nature 2002 416 6880 535 539 10.1038/416535a 11932745

[B42] Iwai A Masliah E Sundsmo MP DeTeresa R Mallory M Salmon DP Saitoh T The synaptic protein NACP is abnormally expressed during the progression of Alzheimer's disease Brain Res 1996 720 1-2 230 234 10.1016/0006-8993(96)00014-5 8782917

[B43] Crews L Tsigelny I Hashimoto M Masliah E Role of Synucleins in Alzheimer's Disease Neurotoxicity Res 2009 16 3 306 317 10.1007/s12640-009-9073-6 PMC2727399 19551456

[B44] Masliah E Rockenstein E Veinbergs I Sagara Y Mallory M Hashimoto M Mucke L β-amyloid peptides enhance alpha-synuclein accumulation and neuronal deficits in a transgenic mouse model linking Alzheimer's disease and Parkinson's disease Proc Natl Acad Sci USA 2001 98 21 12245 12250 10.1073/pnas.211412398 11572944 PMC59799

[B45] Dahlgren KN Manelli AM Stine WB Jr Baker LK Krafft GA LaDu MJ Oligomeric and Fibrillar Species of Amyloid-β Peptides Differentially Affect Neuronal Viability JBiolChem 2002 277 35 32046 32053 10.1074/jbc.M201750200 12058030

[B46] Pike CJ Burdick D Walencewicz AJ Glabe CG Cotman CW Neurodegeneration induced by β-amyloid peptides in vitro: the role of peptide assembly state J Neurosci 1993 13 4 1676 1687 8463843 10.1523/JNEUROSCI.13-04-01676.1993 PMC6576726

[B47] Lambert MP Barlow AK Chromy BA Edwards C Freed R Liosatos M Morgan TE Rozovsky I Trommer B Viola KL Diffusible, nonfibrillar ligands derived from Aβ_1-42 _are potent central nervous system neurotoxins Proc Natl Acad Sci USA 1998 95 11 6448 6453 10.1073/pnas.95.11.6448 9600986 PMC27787

[B48] Lacor PN Buniel MC Furlow PW Sanz Clemente A Velasco PT Wood M Viola KL Klein WL Aβ Oligomer-Induced Aberrations in Synapse Composition, Shape, and Density Provide a Molecular Basis for Loss of Connectivity in Alzheimer's Disease J Neurosci 2007 27 4 796 807 10.1523/JNEUROSCI.3501-06.2007 17251419 PMC6672917

[B49] Shankar GM Bloodgood BL Townsend M Walsh DM Selkoe DJ Sabatini BL Natural Oligomers of the Alzheimer Amyloid-β Protein Induce Reversible Synapse Loss by Modulating an NMDA-Type Glutamate Receptor-Dependent Signaling Pathway J Neurosci 2007 27 11 2866 2875 10.1523/JNEUROSCI.4970-06.2007 17360908 PMC6672572

[B50] Haass C Selkoe DJ Soluble protein oligomers in neurodegeneration: lessons from the Alzheimer's amyloid β-peptide Nat Rev Mol Cell Biol 2007 8 2 101 112 10.1038/nrm2101 17245412

[B51] Passer B Pellegrini L Russo C Siegel RM Lenardo MJ Schettini G Bachmann M Tabaton M D'Adamio L Generation of an Apoptotic Intracellular Peptide by γ-Secretase Cleavage of Alzheimer's Amyloid βProtein Precursor JAlzheimersDis 2000 2 289 301 10.3233/jad-2000-23-408 12214090

[B52] Scheuner D Eckman C Jensen M Song X Citron M Suzuki N Bird TD Hardy J Hutton M Kukull W Secreted amyloid β-protein similar to that in the senile plaques of Alzheimer's disease is increased in vivo by the presenilin 1 and 2 and APP mutations linked to familial Alzheimer's disease Nat Med 1996 2 8 864 870 10.1038/nm0896-864 8705854

[B53] Salmona M Morbin M Massignan T Colombo L Mazzoleni G Capobianco R Diomede L Thaler F Mollica L Musco G Structural properties of Gerstmann-Straussler-Scheinker disease amyloid protein JBiolChem 2003 278 48 48146 48153 10.1074/jbc.M307295200 12970341

[B54] Brewer GJ Isolation and culture of adult rat hippocampal neurons J Neurosci Meth 1997 71 2 143 155 10.1016/S0165-0270(96)00136-7 9128149

[B55] Klingauf J Kavalali ET Tsien RW Kinetics and regulation of fast endocytosis at hippocampal synapses Nature 1998 394 6693 581 585 10.1038/29079 9707119

[B56] Thais ME Carqueja CL Santos TG Silva RV Stroeh E Machado RS Wahlheim DO Bianchin MM Sakamoto AC Brentani RR Synaptosomal glutamate release and uptake in mice lacking the cellular prion protein Brain Res 2006 1075 1 13 19 10.1016/j.brainres.2005.12.045 16519879

